# LC/ESI/TOF-MS Characterization, Anxiolytic and Antidepressant-like Effects of *Mitragyna speciosa* Korth Extract in Diabetic Rats

**DOI:** 10.3390/molecules27072208

**Published:** 2022-03-28

**Authors:** Lin Chen, Shizao Fei, Opeyemi Joshua Olatunji

**Affiliations:** 1 Department of Phycology, Wuhu Second Peoples Hospital, Wuhu 241001, China; 18155317009@163.com; 2 Department of Neurology, Wuhu Second Peoples Hospital, Wuhu 241001, China; 3 Traditional Thai Medical Research and Innovation Center, Faculty of Traditional Thai Medicine, Prince of Songkla University, Hat Yai 90110, Thailand

**Keywords:** *Mitragyna speciosa*, diabetes mellitus, anxiolytic, antidepressant, oxidative stress

## Abstract

In this study, the attenuative effects of the hydro-alcoholic extract from *Mitragyna speciosa* (MSE) against diabetes-induced anxiety and depression-like behaviors were examined. In addition, UPLC/ESI/TOF-MS analysis was performed to identify the phytochemical nature of MSE. DM was induced using a combination of high fructose/streptozotocin, and the diabetic rats were treated with MSE (50 and 200 mg/kg) for 5 weeks. After treatment, the animals were subjected to a forced swim test, open field test and elevated plus-maze tests. Additionally, proinflammatory cytokines and oxidative stress parameters were evaluated in the brain tissues of the rats. UPLC/ESI/TOF-MS analysis revealed that MSE is abundantly rich in polyphenolic constituents, notably flavonoid and phenolic glycosides. Behavioral tests and biochemical analyses indicated that diabetic rats showed significantly increased anxiety and depressive-like behavioral deficits, brain oxidative stress and pro-inflammatory cytokines levels (IL-1β, IL-6 and TNF-α). Treatment with MSE (50 and 200 mg/kg) significantly attenuated increased blood glucose level, depressive and anxiety-like behaviors in diabetic rats. Additionally, the antioxidant enzymes activities were markedly increased in MSE-treated animals, while TNF-α, IL-1β and IL-6 cytokines were notably suppressed. Taken together, these results suggested that MSE has potentials as antidepressant and anxiolytic-like effects and improves the brain oxido-inflammatory status in diabetic rats.

## 1. Introduction

Diabetes mellitus is a chronic metabolic disease which has evolved over the years as a major global public health issue. Typically, diabetes mellitus is principally depicted by excessive blood glucose concentration (hyperglycemia), together with alterations in lipid, carbohydrate and protein metabolism [1,2]. Out of the three different types of diabetes, type II diabetes mellitus is the most rampant accounting for approximately 90–95% of all global cases [1,3]. Insulin resistance, pancreatic beta cell dysfunction and insufficient insulin production are the characteristics hallmarks of type II diabetes mellitus [3]. Hyperglycemia induces excessive generation of reactive oxygen species, leading to oxidative stress, which forms the pathological and physiological basis for all diabetic complications, including nephropathy, neuropathy, cardiovascular diseases, retinopathy and Alzheimer’s disease [4].

One of the most prevailing diabetes associated comorbidity is brain disorders and psychological problems including depression, anxiety, cognitive dysfunctions and Alzheimer’s disease. In fact, it has been reported that up to 30% of DM patients are suffering from depression [5,6,7]. In addition, there is a cordial relationship between hyperglycemia and depressive disorders, as patients exhibiting psychiatric and psychological disorders such as depression and anxiety are prone to increase risk for developing DM, while depression also increases the risk of metabolic disorders particularly DM [7,8,9]. Several factors, including decrease in neurotransmission, increased oxidative and inflammatory factors as well as decrease in antioxidant defense, have been shown to promote insulin resistance and facilitate DM-induced depression [10,11]. As such, mitigating hyperglycemia induced oxidative and inflammatory damage may obviously be a viable target in the treatment of DM-induced neuropsychiatric comorbidities [7,12].

While significant progress has been made in the development of synthetic antidiabetic drugs, unfortunately, adverse reactions as well as the inability of these drugs to impede or slow down the development of DM associated comorbidity have necessitated the search for effective antidiabetic alternatives. Medicinal plants have been enormously explored for their health promoting roles and low toxicity especially in the treatment of diabetes and its associated complications [4,8,12]. Numerous bioactive components from medical plants have been found as excellent antidiabetic agents through their inhibitory effects on several diabetic related pathways [13].

*Mitragyna speciosa* Korth. (kratom) is an indigenous Southeast Asian plant belonging to the family Rubiaceae [14,15]. Historically, *M. speciosa* is used in folk medicine for treating cough, fever, diabetes, pain, diarrhea, cancer, wound, fatigue, hypertension and as a substitute for opium withdrawal [14,15,16,17]. In addition to the traditional uses of *M. speciosa*, accumulating evidences have illustrated the antioxidant, anti-inflammatory, antiobesity, antinociceptive and cytotoxic activities of *M. speciosa* [18,19,20]. Furthermore, *M. speciosa* extracts have been shown to show excellent anxiolytic and anti-depressant effects in mice using two behavioral experiments (forced swim and the tail suspension tests) [14,21]. Aside from the widely reported indole alkaloids (including mitragynine, speciociliatine, paynantheine and 7-hydroxymitragynine) present in *M. speciosa*, several other polyphenolic constituents, such as isoquercitrin, kaempferol, caffeic acid, chlorogenic acid, rutin, apigenin, quercetin, apigenin-7-glycosides, hyperoside, quercetin-3-galactoside-7-rhamnoside, kaempferol-3-glucoside and epicatecin, have also been reported [14,18,22,23]. Despite the widely reported efficacies of *M. speciosa*, there is lack of scientific evidence relating to its antidiabetic therapeutic value as well as its efficacy against diabetes-induced comorbidity. Hence, this study investigated the effects of *M. speciosa* on anxiety and depression-like behaviors, oxidative and inflammatory status in the brain of fructose/streptozotocin-induced diabetic rats.

## 2. Results

### 2.1. LC-ESI-QTOF-MS Analysis for the Identification of Metabolites in MSE

The UPLC-ESI-MS/MS analysis (negative mode) was applied for secondary metabolites profiling of MSE. As portrayed in Table 1, the analysis identified 193 secondary metabolites belonging to chemically diverse classes of compounds, notably flavonoid, phenolics, flavonoid glycoside, iridoid glycosides and ellagitannins subclasses (Table 1). Among the identified compounds, glycosides accounted for the major constituents tentatively identified in MSE, viz., herbacetin-7-glucoside, eriodictyol 7-(6-trans-*p*-coumaroylglucoside), cynaroside A, Kaempferol 3,7,4′-triglucoside, 6-methoxykaempferol 3,7-bis(3-acetylrhamnoside), tiliroside, neoliquiritin 2″-apioside, among several others; phenolic and phenolic glycosides, viz., isoferuloyl C1-glucuronide, protocatechuic acid-3-glucoside, leonuriside A, dihydroferulic acid 4-*O*-glucuronide, glucocaffeic acid and 5-caffeoylquinic acid; iridoid glucosides, viz., mussaenosidic acid, secoxyloganin, shanzhiside methyl ester, theveside, geniposide and swertiapunimarins; triterpene saponins, viz., cynarasaponin F, licoricesaponin B2, tarasaponin, mabioside C and trachelosperoside A1. In addition, alkaloids including mitragynine, lycoricidinol, 10-hydroxystrictosamide, isomitraphyllic acid (16→1)-β-d-glucopyranosyl ester, reserpiline and Echitovenine; stilbenes (*Z*)-resveratrol 3,4′-diglucoside and (*Z*)-resveratrol 3-(3″-sulfoglucoside); anthocyanins, viz., malvidin 3-(6″-acetylglucoside)-5-glucoside, cyanidin 3-(2″-glucuronosylglucoside), malvidin 3-glucoside-5-(6″-malonylglucoside) and cyanidin 3-(2-glucosyl-6-caffeoylglucoside) were also tentatively identified in MSE. Other compounds, such as acridones, purine nucleosides, hydroxycinnamic acid, catechin and coumarin acids, were also revealed in MSE (Table 1).

### 2.2. Effect of MSE on Blood Glucose

**Figure 1:**
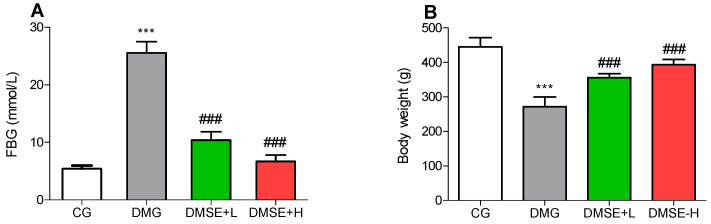
Effect of MSE on (**A**) fasting blood glucose and (**B**) body weight of diabetic rats. Data represent mean ± SD. *** *p* < 0.05 when compared to the control group. ### *p* < 0.05 when compared to the diabetic group.

The results showed that STZ injection leads to hyperglycemia as demonstrated by significant increase in blood glucose concentration of the DMG, MSE-L and DMSE groups compared to the CG (*p* < 0.05; Figure 1A). Treatment with MES (50 and 200 mg/kg) notably reduced the final blood glucose level by 59.4%, and by 74% when compared to the DMG group by the end of the study (*p* < 0.05; Figure 1A).

### 2.3. Effect of MSE on Body Weight

The changes in the initial to the final body weight were evaluated. As shown in Figure 1B, major differences were observed between the body weight of the rats in the DMG group and the CG group. The DMG group showed a 39% decrease in their body weight when juxtaposed with the CG group. After treatment with MSE (50 and 200 mg/kg), the body weight of the treated animals was significant increased (Figure 1B; *p* < 0.05). 

### 2.4. Effect of MSE on Depressive-like Behaviors

**Figure 2:**
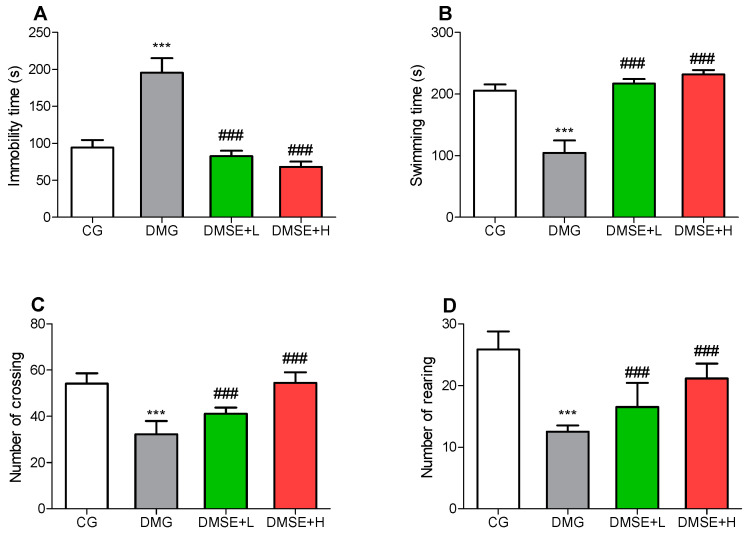
Effect of MSE on (**A**) immobility, (**B**) swimming time in the forced swimming test, (**C**) number of crossing and (**D**) number of rearing in the open field test in diabetic rats. Data represent mean ± SD. *** *p* < 0.05 when compared to the control group. ### *p* < 0.05 when compared to the diabetic group.

DM induced significant increase in the immobility time of the DMG when compared to the CG group in the FST (Figure 2A; *p* < 0.05). However, this effect was markedly decreased in the MSE treated groups compared to the DMG group (Figure 2A; *p* < 0.05). Additionally, the results also revealed significant differences in the swimming time of the DMG compared to the CG group. DMG rats spent less time swimming compared to CG rats (*p* < 0.05, Figure 2B), whereas MSE notably increased the swimming time of the treated rats (*p* < 0.05; Figure 2B).

### 2.5. Effect of MSE on Locomotor Activity

As shown in Figure 2, DMG rats showed significantly decreased locomotive activity, including reduced total number of crossings (Figure 2C) and rearing (Figure 2D) in the open field test when compared to the CG group. Contrariwise, MSE dose dependently improved the locomotive activity of the treated rats (Figure 2C,D).

### 2.6. Effect of MSE on Anxiety-like Behavior

**Figure 3:**
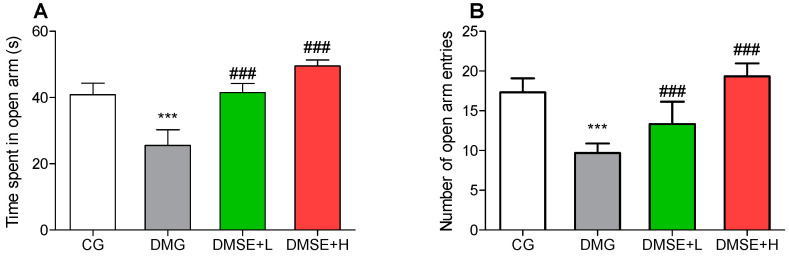
Effect of MSE on the anxiety-like behavior (**A**) time spent in the open arm and (**B**) number of open arm entries in the elevated-plus maze test. Data represent mean ± SD. *** *p* < 0.05 when compared to the control group. ### *p* < 0.05 when compared to the diabetic group.

As shown in Figure 3, DMG rats spent significantly reduced time in the open arms (Figure 3A) and the number of entries into the open arms (Figure 3B) when compared to the CG (*p* < 0.05). MSE induced a significant increase in the time spent in open arms as well as entries into the open arms (*p* < 0.05; Figure 3A,B).

### 2.7. Effect of MSE on Oxidative Stress Parameters

**Figure 4:**
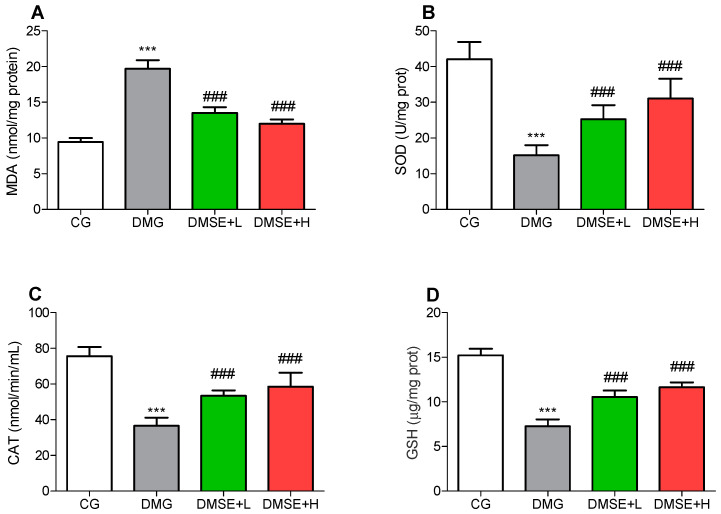
Effect of MSE on oxidative stress parameters in the brain of diabetic rats (**A**) MDA, (**B**) SOD, (**C**) CAT and (**D**) GSH levels. Data represent mean ± SD. *** *p* < 0.05 when compared to the control group. ### *p* < 0.05 when compared to the diabetic group.

Compared with CG, DMG showed a significantly increased level of MDA in the brain, whereas MSE treatment led to a significant decrease in MDA levels compared with the DMG group (Figure 4A; *p* < 0.05). The activities of brain CAT and SOD in the DMG group were obviously lower than the CG group (*p* < 0.05; Figure 4B,C). The activities of CAT and SOD of MSE-treated groups were markedly increased when compared with the DMG group (*p* < 0.05; Figure 4B,C). Additionally, the brain GSH level showed similar trend. The DMG group showed notable decrease in GSH level in comparison with the CG group. Contrariwise, MSE treatment significantly increased the brain GSH level in comparison with the DMG group (*p* < 0.05; Figure 4D). 

### 2.8. Effect of MSE on Proinflammatory Parameters

**Figure 5:**
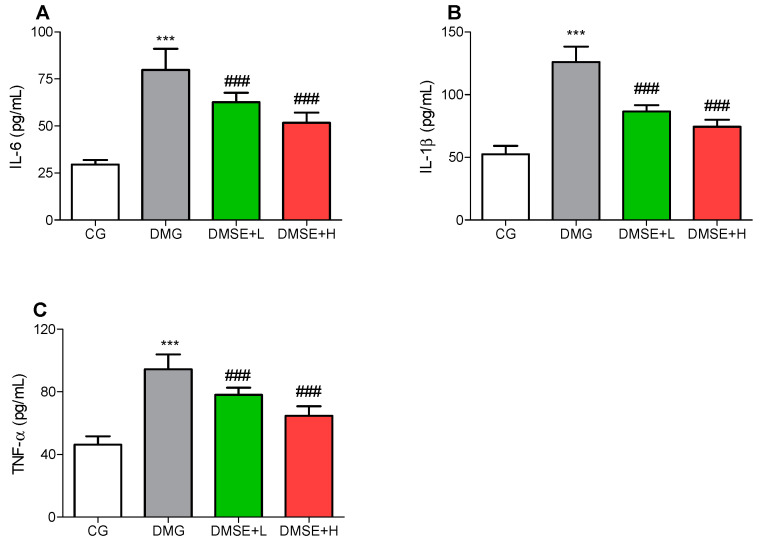
Effect of MSE on proinflammatory cytokines in the brain of diabetic rats (**A**) IL-6, (**B**) IL-1β and (**C**) TNF-α. Data represent mean ± SD. *** *p* < 0.05 when compared to the control group. ### *p* < 0.05 when compared to the diabetic group.

The levels of IL-6, IL-1β and TNF-α were increased significantly in the brain of the DMG group compared with the CG group (Figure 5A–C; *p* < 0.05). MSE significantly alleviated these proinflammatory cytokine levels when compared to the DMG group. Administration of MSE markedly reduced IL-6, IL-1β and TNF-α compared to the DMG group (Figure 5A–C; *p* < 0.05). 

## 3. Discussion

Anxiety and depression constitute a relatively high social hazard and high suicide rate [24] and the incidence of these two psychiatric disorders are 2–3 times higher in diabetic patients when compared to normal people [11]. One of the most sought-after therapeutic approach for the treatment of hyperglycemia and its resulting complications is to keep the blood glucose level at bay, which may subsequently limit the generation of reactive oxygen species and oxidative stress, a major factor implicated in diabetic complications, including DM-induced anxiety and depression [25,26]. Although antidiabetic medicines such as metformin and glibenclamide have been widely successful in the control of blood glucose level, several side effects have limited their success. As such, natural components have been the focus of recent research as alternatives for treating diabetes and its complications [1,4]. *M. speciosa* is well known traditionally and pharmacologically as an antidiabetic, antidepressant, anxiolytic and antioxidant agent. Moreover, *M. speciosa* have shown very strong effects of the brain and central nervous system in several models [14,16]. This study investigated the ameliorative effects of *M. speciosa* extract on depressive and anxiety-like behaviors, as well as oxido-inflammatory status in diabetic rats. This study showed for the first time that *M. speciosa* attenuated anxiety and depressive-like behavior in diabetic rats. These effects seem to be correlated with the hyperglycemic, antioxidant and anti-inflammatory effects of MSE. 

High fructose and STZ-induced DM is a model that elucidates most of the underlying mechanism associated with DM, including insulin resistance, β cells dysfunction and reduced insulin availability, which has been grossly implicated in DM-induced cognitive dysfunction [27]. Furthermore, the brain is one of the major sites of insulin action, as such insulin resistance ultimately leads to neuronal cell death causing dementia, Alzheimer’s disease and depression [28,29]. In this study, DM was induced using a combination of high fructose and STZ, resulting in severe hyperglycemia, and obvious body weight decrease. The inability of the β cells to secrete insulin in DM increases catabolism and gluconeogenesis, resulting in reduced body weight due to wasting of fat storage [7,30]. The increment in blood glucose level and subsequent weight reduction in the diabetic rats were restored upon treatment with MSE. 

The results from this study revealed that DM led to characteristic anxiety and depressive-like behaviors, as indicated by the increase in immobility duration (FST), decrease in entries and time spent in the open arms (EPMT) as well as reduction in the number of crossing and rearing (OFT), which were in corroboration with previous studies on DM-induced anxiety and depression [8,26,31,32]. Metabolic disorders, including diabetes and obesity, have been prominently associated a number of psychological problems particularly major depressive disorders and anxiety, and hyperglycemia is a prevalent factor that can modulate the onset, progression and deterioration of depression and anxiety [33]. MSE treatment notably decreased the duration of immobility in the FST. In addition, MSE administration significantly alleviated anxiety-like behavior in the EPM task by increasing the number of entries and time spent in the open arms. 

Indeed, hyperglycemia has been widely reported to promote oxidative stress, which initiates the onset, progression and deterioration of depression [34,35]. Brain oxidative damage is manifested by reduced antioxidant enzyme activities including SOD, CAT, GSH and increased lipid peroxidation (MDA). SOD, GSH and CAT are the frontline enzymes that initiates cellular protective effects against ROS and oxidative stress element [7,36,37]. In the results presented here, SOD, GSH and CAT activities were significantly decreased, and the MDA level increased significantly in brain structures of the DMG group, which corroborates with previous reports [8,26,38]. Earlier studies have suggested that antidepressant agents could ameliorate oxidative stress, upregulate antioxidant defense, thus restoring redox homeostasis, leading to the reversal of DM-induced anxiety and depressive-like behaviors [25,38,39]. The administration of MSE markedly increase CAT, SOD and GSH activities, as well as decrease the levels of MDA in the brain tissues of treated diabetic rats.

In addition to oxidative stress, numerous studies have reported that neuroinflammation plays a vital role in diabetes-induced depressive and anxiety owing to increased levels of proinflammatory cytokines. DM is associated with neuroinflammation, which may lead to damages or even death of brain neurons. Jawale et al. reported that DM increased the levels of TNF-α in the brain, which mediated anxiety and depressive-like behavior [40,41]. Inflammatory process in DM is a by-product of excessive generation of reactive species and increased oxidative stress, leading to the trigger of NF-κB signaling and subsequent increase in proinflammatory genes [25]. In agreement with previous studies, the level of proinflammatory cytokines (TNF-α, IL-1β and IL-6) was significantly increased in the brain tissues of DMG rats, suggesting neuroinflammation, whereas treatment with MSE attenuated the increased levels of these inflammatory cytokines, suggesting that MSE presents antidepressant-like and anxiolytic effects that may be partly dependent on its antioxidant and anti-inflammatory effects. 

The bioactivity of any natural product especially medicinal plant is largely dependent on the nature and composition of the compounds present in such plant. The existence of polyphenolic compounds in several plant extracts have been reported to be largely responsible for numerous biological activities, notably antioxidant, antidiabetic, antidepressant and anti-inflammatory properties [12,42,43]. In *M. speciosa*, indole alkaloids have been extensively identified as one of the major taxonomic markers of the plant and these compounds have been widely believed to be responsible for the effect of the plant on the brain, pain perception and other activities [44]. However, recent reports have indicated that the presence of other compounds, especially polyphenolic constituents in *M. speciosa*, which strengthens the notion that several other bioactive constituents besides indole alkaloids seems to contribute to bioactive properties of the plant [18,23]. The results obtained from the QTOF-MS analysis of MSE indicated the presence of huge amounts of compounds, ranging from flavonoids, flavonoid glycosides, phenolics, alkaloids, terpene glycosides, stilbenoids and iridoid glycosides. Polyphenolic compounds, especially flavonoids and phenolics, have been reported to be responsible for several antidiabetic and antidiabetic complication effects. The antidiabetic and antidepressant profile of MSE is obviously supported by its chemical constituents. For instance, isoliquiritigenin attenuated DM-induced renal and aortic injury, and displayed significant neuroprotective and antidepressant effects via its effects of oxidative stress and inflammation [45,46,47]. Silvestro et al. reported that quercetin displayed antidepressant-like actions due to its antioxidant, anti-inflammatory and neuroprotective effects in several animal models [48]. In addition, esculetin demonstrated antidiabetic and antidepressant-like properties in DM-induced hepatorenal oxidative damage and LPS-induced neuroinflammatory and depressive-like behavior in mice through its inhibition of oxidative and inflammatory pathways [49,50]. In another report, swertiamarin attenuated hyperglycemia, nephropathy and oxidative stress in DM models [51,52]. Taken together, the synergistic association between all the various groups of compounds in MSE acting on multiple pathways could have collectively exerted the antidiabetic, antidepressant and anxiolytic effects observed in this study. This results further supported the involvement of other bioactive compounds other than indole alkaloids in the bioactivity of kratom plants [18].

## 4. Materials and Methods

### 4.1. Preparation of M. speciosa Extract

Fresh kratom leaves were purchased from a local garden at Nakhon Si Thammarat Province, Thailand. The plant specimen collected was authenticated at the Faculty of Traditional Thai Medicine, Prince of Songkla University, Hat Yai, Thailand and the specimen was preserved at the herbarium of the faculty (Voucher number; KRA-001-22). The dried and powdered kratom leaves were extracted using 80% methanol (*w*/*v*:1:10) in a shaker for 24 h. The extract was filtered, and the leaves mass was subjected to two other rounds of extraction using the same condition as earlier stated. The combined extract was evaporated using a rotary evaporator to approximately 30% of the initial volume. The remaining extract solution was refrigerated at 4 °C overnight. Thereafter, the solution was decanted, centrifuged and lyophilized to obtain a hydroscopic brown colored powder (MSE), which was stored at 4 °C until use.

### 4.2. Metabolite Profiling Using Ultra-High-Performance Liquid Chromatography Coupled to Electrospray Time-of-Flight Tandem Mass Spectrometry (UPLC/ESI/TOF-MS)

The secondary metabolites in MSE were profiled using UHPLC-ESI-QTOF-MS. MSE was dissolved in 50% methanol solution, filtered and the solution obtained after filtration was subjected to the analysis using previously described parameters [53].

### 4.3. Animals and Experimental Design

Six-week-old male Sprague Dawley (SD) rats (140 ± 20 g) were raised under standard conditions (temperature of 22 ± 2 °C, 65 ± 5% relative humidity and a natural photo period of 12 h light/dark cycle). The experimental protocol adhered to the instructions stipulated by Guide for the Care and Use of Laboratory Animals, National Institutes of Health, and approved by the Animal Ethics Committee of The Second Wuhu Second Peoples Hospital (ethics approval number: WheyLLWYH-2021-0908). All the rats were acclimatized for one week on normal rat chow and water ad libitum. After a week of adaptation, the rats were randomly divided into four treatment groups (6 rats/group) as follows: control group (CG): treated with normal saline; diabetic model group (DMG): treated with normal saline; diabetic + MSE-L (DMSE-L): diabetic rats treated with 50 mg/kg MSE; diabetic + MSE-H (DMSE-H): diabetic rats treated with 200 mg/kg MSE. 

### 4.4. Diabetes Induction

Firstly, the diabetic groups (DMG, DMSE-L and DMSE-H) were given 30% fructose solution ad libitum in lieu of normal tap water for four weeks to induce insulin resistance, while the CG group received normal tap water during the same period. Thereafter, DM was induced in overnight-fasted fructose-fed rat groups by intraperitoneal injection of streptozotocin (STZ, 35 mg/kg) solubilized in sodium citrate buffer solution (pH 4.5), while the CG rats were injected with the same volume of sodium citrate buffer. The STZ injected rats were give 5% glucose solution for 24 h in other to avoid possible hypoglycemic state due to STZ injection. The fasting blood glucose (FBG) concentration of all the animals was measured after 3 days of STZ administration with a portable Accu check guide glucometer (Roche Diabetes Care, Mannheim, Germany) and rats with blood glucose concentration >13.88 mmol/L (250 mg/dL) were adjudged as diabetic. MSE was administered to DMSE-L and DMSE-H at 50 and 200 mg/kg, respectively, via oral gavage, while normal saline was administered to the CG and DMG groups daily for five weeks. The dose selection of MSE was based on previous studies [18,54,55,56]. Periodic measurement of the blood glucose concentration and body weights were evaluated.

### 4.5. Open Field Test

The test was performed using a rectangular wooden open field with dimensions 40 × 50 × 65 cm, and the floor of the field was divided into six concentric units. The rats were placed in the middle of the field. The rats’ ambulation including the total number of crossings with all four paws and the number of rearing was recorded for 5 min. A trained observer blinded to the treatment protocol evaluated the total number of cross and rearing of each animal.

### 4.6. Elevated Plus Maze Test

The EPMT was made up of two open and two closed arms with (50 × 10 cm) perpendicularly arranged and linked by a central sheath 50 cm high from the floor. Each rat was gradually positioned in the central square of the apparatus, facing the open arms and allowed to explore for 300 s. The time spent and number of entries into the open arms was measured during a 5 min period. In other to eliminate any olfactory cues, the apparatus was cleaned with 70% alcohol between each examination.

### 4.7. Forced Swimming Test

**Figure 6:**
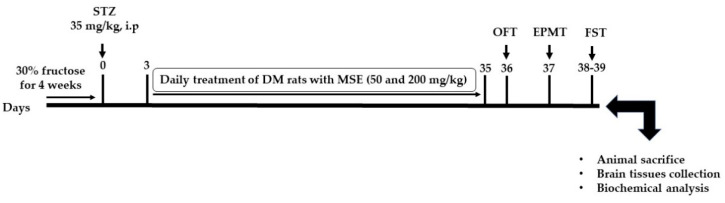
Experimental schematic diagram.

The forced swimming test apparatus consisted of a cylindrical shaped glass container (40 × 30 cm) filled with water (23 ± 2 °C) to a depth of 30 cm. The rats were initially forced to swim for 15 min. After 24 h, the rats were subjected to the same procedure for 5 min, during the period the immobility and swimming time were manually recorded. Immobility time was taken as the period where no activity was initiated by the rats except for movements that kept the rat’s head above water. A trained observer blinded to the treatment protocol evaluated the swimming and immobility time of each animal. The experimental timeline is shown in Figure 6. 

### 4.8. Sacrifice and Sample Collection

The rats were fasted for 12 h and humanely sacrificed by euthanizing with sodium thiopental (150 mg/kg). The brain tissues were dissected from the rats, cleaned with physiological saline solution and instantly fixed in 10% neutral buffered formalin solution for further histopathological analysis. Another portion of the brain tissues were preserved at −70 °C for further analysis.

### 4.9. Analysis of Oxidative and Inflammatory Biomarkers

The isolated whole brain tissues were homogenized using potassium phosphate buffer (pH 6.5). After centrifugation (6000× *g* for 30 min at 4 °C), the supernatant collected was used for evaluating glutathione (GSH), superoxide dismutase (SOD), catalase (CAT) and malondialdehyde (MDA) levels using assay kits from Jiancheng Biotechnology, Nanjing, China. Likewise, proinflammatory cytokine levels; interleukin 6 (IL-6), interleukin 1β (IL-1β) and tumor necrosis factor alpha (TNF-α) were measured using ELISA kits obtained from Abcam, Cambridge, UK, following the manufacturers procedures.

### 4.10. Statistical Analysis

Statistical analysis of the data was performed using GraphPad Prism software (version 5.0, GraphPad Software, Inc. San Diego, CA, USA) and normal distribution of the data was checked by the Kolmogorov–Smirnov test. Data displayed mean ± standard deviation and were analyzed by one way ANOVA followed by Tukey’s post-hoc test. *p* < 0.05 indicated statistical significance.

## 5. Conclusions

The findings from this study demonstrated that the administration of MSE attenuated depressive and anxiety-like behaviors induced by fructose/STZ diabetic rats. The alleviative effects of MSE were mediated through the reduction of oxidative stress and neuroinflammation (revealed by decreased MDA, IL-6, IL-1β and TNF-α levels) in the brain of the treated diabetic rats. Furthermore, LC-ESI-MS analysis of MSE revealed the overwhelming majority polyphenolic compounds, including phenolic, flavonoids, terpenoids, iridoids and their glycosidic bound forms. Overall, the results highlighted the potential biological effects of MSE in the treatment of diabetes and its associated comorbidity. 

## Figures and Tables

**Table 1:** Constituents tentatively identified in MSE extract using LC-ESI-QTOF-MS analysis.

No.	RT (min)	Mass (*m*/*z*)	Elemental Composition	DB Diff (ppm)	Tentative Metabolite Identity
1	1.639	426.0767	C_19_H_16_F_2_O_9_	−1.01	Diflunisal phenolic glucuronide
2	1.662	252.0953	C_8_H_16_N_2_O_7_	1.81	Cycasin
3	1.719	556.1377	C_31_H_24_O_10_	−1.39	Dianhydroaurasperone C
4	1.725	370.09	C_16_H_18_O_10_	0.01	Isoferuloyl C1-glucuronide
5	1.729	654.2207	C_36_H_34_N_2_O_10_	1	Citbismine F
6	1.778	128.0467	C_6_H_8_O_3_	5.11	Osmundalactone
7	1.787	518.1835	C_19_H_34_O_16_	2.32	Ciceritol
8	1.81	696.1922	C_31_H_36_O_18_	−2.9	Malvidin 3-(6″-acetylglucoside)-5-glucoside
9	1.831	366.1128	C_18_H_22_O_6_S	2.57	4-Hydroxyestrone sulfate
10	1.885	470.088	C_20_H_22_O_11_S	0.55	(*Z*)-Resveratrol 3-(3″-sulfoglucoside)
11	1.907	238.1043	C_9_H_18_O_7_	4.07	(x)-1,2-Propanediol 1-*O*-β-d-glucopyranoside
12	1.922	291.095	C_11_H_17_NO_8_	1.32	Sarmentosin epoxide
13	2.216	135.0544	C_5_H_5_N_5_	0.87	Adenine
14	2.35	636.0096	C_22_H_20_O_18_S_2_	−0.8	Isoscutellarein 4′-methyl ether 8-(2″,4″-disulfatoglucuronide)
15	2.354	408.1068	C_19_H_20_O_10_	−2.76	Khellol glucoside
16	2.75	283.091	C_10_H_13_N_5_O_5_	2.51	Crotonoside
17	4.43	406.1469	C_17_H_26_O_11_	1.42	Shanzhiside methyl ester
18	5.101	390.1158	C_16_H_22_O_11_	1.14	Theveside
19	5.284	402.1157	C_17_H_22_O_11_	1.26	4-Hydroxy-5-(3′,5′-dihydroxyphenyl)-valeric acid-*O*-glucuronide
20	5.311	316.0787	C_13_H_16_O_9_	2.34	Protocatechuic acid-3-glucoside
21	5.379	332.1101	C_14_H_20_O_9_	1.91	Leonuriside A
22	5.453	464.0948	C_21_H_20_O_12_	1.48	Herbacetin 7-glucoside
23	5.529	356.073	C_15_H_16_O_10_	1.51	Caffeoyl C1-glucuronide
24	6.221	300.0839	C_13_H_16_O_8_	1.91	Salicylic acid β-d-glucoside
25	6.417	360.105	C_15_H_20_O_10_	1.89	Glucosyringic acid
26	6.435	376.1363	C_16_H_24_O_10_	1.72	Mussaenosidic acid
27	6.456	436.1	C_20_H_20_O_11_	1.22	Taxifolin 3-arabinoside
28	6.722	112.0159	C_5_H_4_O_3_	0.99	Pyromeconic acid
29	7.546	404.1315	C_17_H_24_O_11_	1.02	Secoxyloganin
30	7.663	286.0685	C_12_H_14_O_8_	1.4	Uralenneoside
31	7.665	342.0947	C_15_H_18_O_9_	1.23	Glucocaffeic acid
32	7.813	406.2193	C_19_H_34_O_9_	2.53	(*3S*,*5R*,*6R*,*7E*,*9S*)-Megastigman-7-ene-3,5,6,9-tetrol 9-*O*-β-d-glucopyranoside
33	8.017	376.1365	C_16_H_24_O_10_	1.3	Loganic acid
34	8.135	596.1521	C_30_H_28_O_13_	1.44	Eriodictyol 7-(6-*trans*-*p*-coumaroylglucoside)
35	8.259	342.0948	C_15_H_18_O_9_	0.88	Glucocaffeic acid
36	8.286	444.199 C	C_21_H_32_O_10_	1.26	Cynaroside A
37	8.879	446.1419	C_19_H_26_O_12_	1.16	Lucuminic acid
38	8.884	418.1461	C_18_H_26_O_11_	3.32	2-C-Methyl-d-erythritol 1-*O*-β-d-(6-*O*-4-hydroxybenzoyl)glucopyranoside
39	8.928	402.1893	C_19_H_30_O_9_	−0.8	d-Linalool 3-(6″-malonylglucoside)
40	8.976	372.105	C_16_H_20_O_10_	1.67	Dihydroferulic acid 4-*O*-glucuronide
41	9.166	354.0948	C_16_H_18_O_9_	0.92	5-Caffeoylquinic acid
42	9.263	772.205	C_33_H_40_O_21_	1.58	Kaempferol-3,7,4′-triglucoside
43	9.309	290.0785	C_15_H_14_O_6_	1.81	Epifisetinidol-4α-ol
44	9.322	452.1312	C_21_H_24_O_11_	1.47	7-Hydroxybutylidenephthalide 7-(6-malonylglucoside)
45	9.364	692.1939	C_32_H_36_O_17_	1.97	6-Methoxykaempferol 3,7-bis(3-acetylrhamnoside)
46	9.379	390.1159	C_16_H_22_O_11_	0.74	Deacetyl asperulosidic acid
47	9.435	426.173	C_17_H_30_O_12_	1.64	3-Methylbutanoyl-6-*O*-α-d-glucopyranosyl-β-d-fructofuranoside
48	9.522	625.1409	C_27_H_29_O_17_	−0.76	Cyanidin 3-(2″-glucuronosylglucoside)
49	9.533	306.0732	C_15_H_14_O_7_	2.57	Epigallocatechin
50	9.576	326.0997	C_15_H_18_O_8_	1.48	*o*-Coumaric acid-β-d-glucoside
51	9.584	392.1312	C_16_H_24_O_11_	1.59	Caryoptosidic acid
52	9.796	374.1207	C_16_H_22_O_10_	1.54	Swertiamarin
53	9.81	330.0944	C_14_H_18_O_9_	2.02	3′-Glucosyl-2′,4′,6′-trihydroxyacetophenone
54	9.841	742.231	C_33_H_42_O_19_	1.43	Naringin 4′-glucoside
55	9.91	722.1473	C_35_H_30_O_17_	1.43	Thonningianin B
56	9.976	460.1575	C_20_H_28_O_12_	1.27	Methyl salicylate *O*-[rhamnosyl-(1→6)-glucoside]
57	9.977	528.1446	C_30_H_24_O_9_	−4.83	Mahuangnin D
58	10.129	756.2101	C_33_H_40_O_20_	1.64	Kaempferol 3-rutinoside-4′-glucoside
59	10.14	316.1152	C_14_H_20_O_8_	1.80	3,4-Dihydroxyphenylethyl alcohol glucoside
60	10.196	178.0262	C_9_H_6_O_4_	2.35	Aesculetin
61	10.219	326.1002	C_15_H_18_O_8_	−0.06	*cis*-β-d-Glucosyl-2-hydroxycinnamate
62	10.329	866.2047	C_45_H_38_O_18_	1.34	Cinnamtannin A1
63	10.4	402.152	C_18_H_26_O_10_	1.41	Benzyl *O*-[arabinofuranosyl-(1→6)-glucoside]
64	10.401	516.1443	C_29_H_24_O_9_	−4.35	Thelephantin A
65	10.403	578.142	C_30_H_26_O_12_	0.71	Apigenin 7-(4″-*E*-*p*-coumarylglucoside)
66	10.566	430.1468	C_19_H_26_O_11_	1.58	Bungeiside C
67	10.609	566.1839	C_23_H_34_O_16_	1.32	7-Glucosyl-11-methylodeoside
68	10.635	1154.2717	C_60_H_50_O_24_	−2.14	Cinnamtannin A2
69	10.759	386.121	C_17_H_22_O_10_	0.78	3′-*O*-β-glucopyranosyl plumbagic acid
70	10.796	458.1417	C_20_H_26_O_12_	1.64	7-Hydroxy-4-methylphthalide *O*-[arabinosyl- (1→6)-glucoside]
71	10.882	886.2728	C_39_H_50_O_23_	1.67	Reiniose E
72	10.882	886.2728	C_16_H_18_O_8_	1.4	1,2,4-Trihydroxynaphthalene-4-glucoside
73	10.986	388.1364	C_17_H_24_O_10_	1.35	Geniposide
74	11.042	490.1681	C_21_H_30_O_13_	1.15	Phloroacetophenone 6′-[xylosyl-(1→6)- glucoside]
75	11.057	630.1941	C_31_H_34_O_14_	1.23	(*R*)-Rutaretin 1′-(6″-sinapoylglucoside)
76	11.156	390.1522	C_17_H_26_O_10_	0.98	Todatriol glucoside
77	11.161	386.1935	C_19_H_30_O_8_	0.93	Roseoside
78	11.276	630.1943	C_31_H_34_O_14_	0.87	(*R*)-Rutaretin 1′-(6″-sinapoylglucoside)
79	11.345	520.1785	C_22_H_32_O_14_	1.30	Swertiapunimarin
80	11.372	590.1388	C_38_H_22_O_7_	−3.75	4′-Hydroxyanigorootin
81	11.372	582.1731	C_30_H_30_O_12_	1.15	Epicatechin 3-*O*-(3-*trans*-cinnamoyl-β-d-allopyranoside)
82	11.392	307.0686	C_14_H_13_NO_7_	1.82	Lycoricidinol
83	11.398	246.0886	C_14_H_14_O_4_	2.65	Columbianetin
84	11.408	648.2043	C_31_H_36_O_15_	1.73	Embigenin 2″-(2′″-acetylrhamnoside)
85	11.412	404.1316	C_17_H_24_O_11_	0.73	Secoxyloganin
86	11.425	704.2306	C_34_H_40_O_16_	1.49	Amorphigenin *O*-vicianoside
87	11.501	462.1724	C_20_H_30_O_12_	2.87	Verbasoside
88	11.641	344.1464	C_16_H_24_O_8_	1.95	Dihydroconiferin
89	11.665	461.1524	C_19_H_27_NO_12_	2	4-(2-Nitroethyl)phenyl primeveroside
90	11.668	444.1626	C_20_H_28_O_11_	1.23	Sibiricaphenone
91	11.734	450.1158	C_21_H_22_O_11_	0.90	Sinensin
92	11.882	864.1891	C_45_H_36_O_18_	1.21	Pavetannin B2
93	12.058	356.1105	C_16_H_20_O_9_	0.61	Gentiopicrin
94	12.5	434.0844	C_20_H_18_O_11_	1.20	Quercetin 3-β-l-arabinopyranoside
95	12.539	400.1363	C_18_H_24_O_10_	1.51	3′-*O*-β-Glucopyranosyl plumbagic acid methyl ester
96	12.788	372.1049	C_16_H_20_O_10_	1.98	Veranisatin C
97	12.807	404.1315	C_17_H_24_O_11_	0.79	Theviridoside
98	12.867	370.1256	C_17_H_22_O_9_	2.07	Perilloside E
99	12.991	414.1517	C_19_H_26_O_10_	2.18	Ptelatoside A
100	13.052	394.1832	C_17_H_30_O_10_	1.67	*cis*-3-Hexenyl β-primeveroside
101	13.102	514.194	C_26_H_30_N_2_O_9_	2.28	10-Hydroxystrictosamide
102	13.247	346.1623	C_16_H_26_O_8_	1.27	Villoside
103	13.266	739.2082	C_33_H_39_O_19_	0.46	Malvidin 3-glucoside-5-(6″-malonylglucoside)
104	13.381	224.1405	C_13_H_20_O_3_	3.14	Vomifoliol
105	13.966	476.1885	C_21_H_32_O_12_	1.75	Kanokoside A
106	14.095	368.11	C_17_H_20_O_9_	1.99	3-*O*-Caffeoyl-4-*O*-methylquinic acid
107	14.375	436.1726	C_22_H_28_O_9_	1.79	Icaride A2
108	14.491	740.173	C_39_H_32_O_15_	1.55	3″,6″-Di-*O*-*p*-coumaroyltrifolin
109	15.137	384.1414	C_18_H_24_O_9_	1.76	Tenuifoliside D
110	15.901	516.2101	C_26_H_32_N_2_O_9_	1.38	Isomitraphyllic acid (16→1)-β-d-glucopyranosyl ester
111	15.921	464.0948	C_21_H_20_O_12_	1.39	Robinetin 7-glucoside
112	15.978	452.1102	C_24_H_20_O_9_	1.07	Epigallocatechin 3-*O*-*p*-coumarate
113	16.185	348.1777	C_16_H_28_O_8_	2.19	Foeniculoside VIII
114	16.234	304.0578	C_15_H_12_O_7_	1.6	2′,3,5,6′,7-Pentahydroxyflavanone
115	16.288	594.1582	C_27_H_30_O_15_	0.4	Isoorientin 7-*O*-rhamnoside
116	16.29	582.194	C_27_H_34_O_14_	1.45	10-Acetoxyligustroside
117	16.296	1064.2994	C_48_H_56_O_27_	1.44	Capilliposide I
118	16.329	464.2259	C_21_H_36_O_11_	−0.22	Linalool 3,7-oxide β-primeveroside
119	16.334	460.1583	C_20_H_28_O_12_	−0.57	Aplopaeonoside
120	16.335	576.1261	C_30_H_24_O_12_	1.17	Epicatechin-(2β→5,4β→6)-entepicatechin
121	16.343	592.1571	C_31_H_28_O_12_	1.62	8,8′-Methylenebiscatechin
122	16.366	552.1838	C_26_H_32_O_13_	0.89	(*Z*)-Resveratrol 3,4′-diglucoside
123	16.417	754.1892	C_40_H_34_O_15_	0.71	Chrysoeriol 7-(3″,6″-di-(*E*)-*p*-coumaroylglucoside)
124	16.424	522.162	C_25_H_30_O_12_	22.4	Melampodinin
125	16.435	484.1363	C_25_H_24_O_10_	1.31	Silidianin
126	16.479	518.1992	C_23_H_34_O_13_	1.37	Jioglutoside B
127	16.487	612.2029	C_28_H_36_O_15_	4.1	Neohesperidin dihydrochalcone
128	16.518	410.1578	C_20_H_26_O_9_	−0.32	Yadanziolide C
129	16.525	448.1001	C_21_H_20_O_11_	1.03	Petunidin-3-*O*-arabinoside
130	16.55	1048.3048	C_48_H_56_O_26_	1.11	Quercetin 3-rhamnosyl-(1→6)-[rhamnosyl-(1→2)-(3″-(*E*)-*p*-coumaroylgalactoside)]-7-Rhamnoside
131	16.575	520.1938	C_26_H_32_O11	1.18	Tetracentronside B
132	16.621	580.2147	C_28_H_36_O_13_	1.58	(+)-7-epi-Syringaresinol 4′-glucoside
133	16.655	420.1634	C_18_H_28_O_11_	−0.61	Lamioside
134	16.663	412.1988	C_23_H_28_N_2_O_5_	2.52	Reserpiline
135	16.766	528.1625	C_27_H_28_O_11_	1.34	Tremulacin
136	16.821	548.2251	C_28_H_36_O_11_	1.19	Bruceantin
137	16.822	504.1839	C_22_H_32_O_13_	0.84	(*S*)-Multifidol 2-[apiosyl-(1→6)-glucoside]
138	16.85	448.1365	C_22_H_24_O_10_	1.04	Licoagroside D
139	16.927	436.1155	C_24_H_20_O_8_	0.76	(−)-Epigallocatechin 3-cinnamate
140	16.934	498.1517	C_26_H_26_O_10_	1.79	Dukunolide B
141	16.946	772.1847	C_36_H_36_O_19_	0.49	Cyanidin 3-(2-glucosyl-6-caffeoylglucoside)
142	16.949	902.2468	C_42_H_46_O_22_	1.4	Kaempferol 3-caffeylrobinobioside-7-Rhamnoside
143	16.978	342.0732	C_18_H_14_O_7_	2.09	Iriskashmirianin
144	16.998	828.4492	C_42_H_68_O_16_	1.9	Centellasaponin B
145	17.017	436.1362	C_21_H_24_O_10_	1.75	Phlorhizin
146	17.025	317.0665	C_16_H_13_O_7_	−1.09	Petunidin
147	17.029	942.4798	C_47_H_74_O_19_	2.77	Phytolaccoside I
148	17.052	466.1259	C_25_H_22_O_9_	1.12	Silymonin
149	17.067	756.1896	C_36_H_36_O_18_	0.68	Kaempferol 3-glucoside-7-*p*-coumarylglucoside
150	17.11	856.4445	C_43_H_68_O_17_	1.35	Laxogenin 3-*O*-{*O*-β-d-xylopyranosyl-(1→4)-*O*-[α-l-arabinopyranosyl-(1→6)-β-d-glucopyranoside
151	17.114	766.4492	C_41_H_66_O_13_	1.48	Soyasaponin IV
152	17.135	532.2311	C_28_H_36_O_10_	−0.47	Nomilinic acid
153	17.232	812.4543	C_42_H_68_O_15_	1.86	Kudzusaponin SA1
154	17.237	956.4974	C_48_H_76_O_19_	0.76	Chikusetsusaponin V
155	17.293	550.1678	C_26_H_30_O_13_	1.45	Neoliquiritin 2″-apioside
156	17.331	194.0574	C_10_H_10_O_4_	2.81	Isoferulic acid
157	17.356	780.4285	C_41_H_64_O_14_	1.37	Cynarasaponin F
158	17.411	808.4241	C_42_H_64_O_15_	0.58	Licoricesaponin B2
159	17.411	926.4899	C_47_H_74_O_18_	−2.55	Tarasaponin I
160	17.425	842.4645	C_43_H_70_O_16_	2.28	Aspafilioside B
161	17.487	750.4547	C_41_H_66_O_12_	1.03	Scabioside B
162	17.514	666.3966	C_36_H_58_O_11_	1.98	Arjunglucoside I
163	17.548	878.3963	C_44_H_62_O_18_	−3.03	Hancoside A
164	17.559	638.3654	C_34_H_54_O_11_	1.91	26-Glucosyl-1,3,11,22-tetrahydroxyergosta-5,24-dien-26-oate
165	17.619	422.2144	C_19_H_34_O_10_	1.84	1-Octen-3-yl primeveroside
166	17.656	396.2142	C_21_H_32_O_7_	1.64	Isopetasoside
167	17.748	288.0622	C_15_H_12_O_6_	4.12	3,4,2′,4′,α-Pentahydroxychalcone
168	17.748	446.2143	C_21_H_34_O_10_	1.95	Dendroside G
169	17.796	302.0416	C_15_H_10_O_7_	3.59	Quercetin
170	17.8	396.2044	C_23_H_28_N_2_O_4_	1.38	Echitovenine
171	17.8	530.2511	C_29_H_38_O_9_	0.99	Angeloylgomisin Q
172	17.813	516.2356	C_28_H_36_O_9_	0.73	Kupitengester 4
173	17.822	398.2201	C_23_H_30_N_2_O_4_	1.04	Mitragynine
174	17.918	648.3862	C_36_H_56_O_10_	1.73	2α-Hydroxygypsogenin 3-*O*-β-d-glucoside
175	17.992	792.4281	C_42_H_64_O_14_	1.86	Mabioside C
176	18.112	680.3765	C_36_H_56_O_12_	0.99	Trachelosperoside A1
177	18.133	208.0731	C_11_H_12_O_4_	2.28	6-Methoxymellein
178	18.443	650.4016	C_36_H_58_O_10_	2.16	Lucyoside N
179	18.577	450.2607	C_25_H_38_O_7_	2.27	Laserpitin
180	18.82	632.3912	C_36_H_56_O_9_	1.99	Lucyoside K
181	18.821	796.459	C_42_H_68_O_14_	2.35	Saikosaponin L
182	18.868	192.0781	C_11_H_12_O_3_	2.88	(*R*)-Shinanolone
183	19.065	250.1198	C_14_H_18_O_4_	2.69	Helinorbisabone
184	19.189	252.0992	C_13_H_16_O_5_	2.30	Methyl 3,4,5-trimethoxycinnamate
185	19.514	594.136	C_30_H_26_O_13_	2.22	Tiliroside
186	19.597	256.0728	C_15_H_12_O_4_	2.92	Isoliquiritigenin
187	19.677	222.1611	C_14_H_22_O_2_	3.96	Isokobusone
188	19.678	294.1826	C_17_H_26_O_4_	1.64	Myrsinone
189	20.303	488.3487	C_30_H_48_O_5_	2.97	Uncaric acid
190	20.674	276.1716	C_17_H_24_O_3_	3.36	Moxartenone
191	20.849	292.1665	C_17_H_24_O_4_	3.2	6-Hydroxyshogaol
192	21.329	234.1613	C_15_H_22_O_2_	2.88	Sclerosporin
193	23.648	430.2005	C_24_H_30_O_7_	−3.20	Schisanlignone A

## Data Availability

Not applicable.

## Sample Availability

Samples of the compounds are not available from the authors.
